# Annotations

**Published:** 1908-02-29

**Authors:** 


### ANNOTATIONS.

### The Adulteration of Drugs.

Mr. E. M. Holmes, curator of the Pharmaceutical Society's Museums, recently read a paper before the Society upon the adulteration of drugs. There were several points of interest in Mr. Holmes's communication. His definition of the word adulteration, in so far as it concerns drugs, seems sufficiently precise and comprehensive. He regards it, firstly, as the addition of any foreign material, by whomsoever effected, whereby the natural condition of the drug is interfered with, and its medicinal value is lessened; secondly, as the substitution of a drug of inferior quality for the genuine substance, even if of the same genus. Either of these forms of adulteration renders the action of medicine uncertain. Mr. Holmes is of the opinion that the greater part of drug adulteration occurs at the place of production, and that cases of adulteration by pharmacists are exceedingly rare. He traces much of the responsibility for this evil to the increasing demand by the public for cheap drugs; and with this view we are inclined, to a certain extent, to agree. As to his suggestion that definite legal standards should be laid down for all drugs, we must, of course, acknowledge that this would be a most admirable reform. But we are afraid that many difficulties would present themselves to those responsible for laying down practicable standards of purity and efficiency. Whilst it is desirable, in the interests of the public and of the medical profession, that efficient chemical and pharmacological standards should be established, their enforcement in the case of certain important drugs would prove to be a task of great difficulty. If, nevertheless, the law can be induced to face the obstacles in the way of drug standardisation, we have little doubt that it will adopt Mr. Holmes's suggestion that, in the case of a drug which is used both for medicinal and technical purposes, two standards should be laid down.

### Damages against a Cancer Quack.

At the Manchester Assizes last week, before Mr. Justice Pickford and a special jury, the case of Annie Johnson *v.* Dr. Hall's Hygiene Company was tried. The plaintiff's case was that Hall held himself out as a registered medical practitioner, under the name of Dr. Hall's Hygiene Company, at Pendleton, Manchester. By means of advertisements he claimed to be specially qualified to deal with diseases of women, including tumours and cancer of the breast. The plaintiff consulted him in August 1906 about a small, hard swelling in her breast, wishing to know if it was cancer. The defendant examined it and said: "No, this is not cancer, but congestion. I will give you remedies which will dissolve it." He ordered baths, oils, fomentations, ointments, herbs, mixtures, and a poultice of slippery elm; but in spite of these measures the swelling increased in size, and invaded the axilla. He assured her throughout that it was not cancer, and that no operation was necessary. After a year of this treatment the swelling and pain were so great that the plaintiff was forced to consult a local doctor. An immediate operation was performed, but this was merely palliative, and there was early recurrence of the disease. Dr. Shaw, the only witness called for the defence, admitted that the defendant's treatment was negative and ineffectual. The Judge characterised Hall as a quack doctor, who attacked the medical profession for exposing women for their own gain to revolting examinations and to unnecessary and cruel operations. The jury, after seven minutes' consultation, awarded £600 damages to the plaintiff.

### Medical Inspection of School Children.

The medical inspection of school children has now become a statutory duty, and the lines on which this duty is to develop and the conditions which are to govern it are of the first importance to the medical profession. It is probable that a sound policy in these directions will only be secured as a result of time and experience, but certain principles must necessarily be defined at the outset. Hence it is satisfactory to note that various professional organistions are devoting their best attention to the subject, and it is much to be hoped that there will be produced a practically unanimous view by which the profession as a whole will be guided. Otherwise there is a real danger that divided counsels will mean a loss of the important influence which the profession ought to exert on this subject, and we shall see repeated the unfortunate position which is occupied by medical practitioners in many other departments of public medical work. Now that the Board of Education includes a properly equipped medical department, there is reason to hope that due regard will be paid to the claims of the profession in respect to the responsibilities which will be placed on the shoulders of a number of individual practitioners. But everything must not be left to the central authorities. On the contrary, there must be in the various localities such an appreciation of the circumstances, and so clear a discussion of the issues at stake, that the Board may know exactly what are the views of those upon whom the immediate responsibilities will fall. In some quarters there still appears to remain the opinion that the supervision in any large school area ought to be separated from the acting medical officer of health. But it is a waste of time to discuss this issue, for the contrary view has received official endorsement, and in the meantime at least no change in this respect is possible. Where professional influence is specially needed is in the definition of the duties of those to whom the actual details of inspection will be committed, and in the remuneration to be attached to these duties. Another point of great importance is the separation of the functions of medical examination and treatment. To secure this is essential, and, further, unless care is taken there will be a still further extension of the class who accept medical charity though their circumstances do not justify any such consideration.

